# Functional innate immunity restricts Hepatitis C Virus infection in induced pluripotent stem cell–derived hepatocytes

**DOI:** 10.1038/s41598-018-22243-7

**Published:** 2018-03-01

**Authors:** Anja Schöbel, Kathrin Rösch, Eva Herker

**Affiliations:** Heinrich Pette Institute, Leibniz Institute for Experimental Virology, Hamburg, Germany

## Abstract

Knowledge of activation and interplay between the hepatitis C virus (HCV) and the hosts’ innate immunity is essential to understanding the establishment of chronic HCV infection. Human hepatoma cell lines, widely used as HCV cell culture system, display numerous metabolic alterations and a defective innate immunity, hindering the detailed study of virus-host interactions. Here, we analysed the suitability of induced pluripotent stem cell (iPSC)-derived hepatocyte-like cells (iHLCs) as a physiologically relevant model to study HCV replication *in vitro*. Density gradients and triglyceride analysis revealed that iHLCs secreted very-low density lipoprotein (VLDL)-like lipoproteins, providing a putative platform for *bona fide* lipoviroparticles. iHLCs supported the full HCV life cycle, but in contrast to Huh7 and Huh7.5 cells, replication and viral RNA levels decreased continuously. Following HCV infection, interferon-stimulated gene (ISG)-expression significantly increased in iHLCs, whereas induction was almost absent in Huh7/7.5 cells. However, IFNα-stimulation equally induced ISGs in iHLCs and hepatoma cells. JAK-STAT pathway inhibition increased HCV replication in mature iHLCs, but not in Huh7 cells. Additionally, HCV replication levels where higher in STAT2-, but not STAT1-knockdown iHLCs. Our findings support iHLCs as a suitable model for HCV-host interaction regarding a functional innate immunity and lipoprotein synthesis.

## Introduction

Chronic hepatitis C virus (HCV) infection still remains a major public health problem worldwide, leading to severe secondary liver diseases such as cirrhosis or hepatocellular carcinoma. Current knowledge of molecular mechanisms in HCV-host interaction is often based on *in vitro* experiments using well-established hepatoma cell lines (Huh7 and its derivates). Despite their convenience, those cell lines frequently differ from the *in vivo* state and primary hepatocytes in important aspects regarding metabolic pathways, proliferation, and innate immune response^1,2^. For example, Huh7 cells show an impaired lipoprotein metabolism, as they do not produce *bona fide* very low-density lipoproteins (VLDLs) but apolipoprotein B (ApoB)-containing particles that resemble low-density lipoproteins (LDLs)^3,4^. The HCV life cycle is closely linked to the hepatic lipoprotein metabolism as viral particles associate with lipoproteins, most prominently ApoE, and lipids during maturation to form lipoviroparticles (LVPs)^5^. Accordingly, cell culture-derived HCV particles (HCVcc) produced in Huh7-derived cells show a higher buoyant density compared to *in vivo-* or primary hepatocyte–derived samples, correlating with a lower specific infectivity^3,6^. Intriguingly, production of infectious particles in Huh7-derived cells depends on ApoE but not ApoB expression^7^. Another drawback of using the hepatoma cell lines to study infectious processes is their diminished innate immunity^8–10^.

In order to understand viral persistence, studying the interplay of HCV and the host cells in a physiologically intact model system is thus an important aspect. As access to primary human hepatocytes is limited and their long-term cultivation remains challenging, the creation of induced pluripotent stem cells (iPSCs) opened up possibilities for an alternative model for *in vitro* studies^11,12^. iPSCs provide a robust regenerating source for various cell types and, derived from different donors, enable the analysis of different genetic backgrounds as well as sex dependencies in various disease-related questions^13^. Successful differentiation into functional hepatocyte-like cells (iHLCs) has been described in several reports^14–16^.

Over the last years, iHLC-based cell culture systems have been established for drug toxicity testing^17–19^ as well as for infectivity studies of different pathogens, such as dengue virus, *plasmodium spp*., and the hepatitis B (HBV) - and C-virus (HCV)^20–25^. iHLCs support HCV RNA replication as well as production of infectious viral particles, but the biophysical characteristics of those particles have not been assessed yet. More recently, activation of the interferon-pathway in HCV-infected stem cell-derived hepatocytes was described^26–28^, indicating an intact innate response.

In this study, we examined the functionality of iHLCs to serve as a physiologically relevant model system for HCV *in vitro* studies. Mature iHLCs displayed hepatocyte specific markers as well as metabolic functions. Importantly, lipoproteins secreted from iHLCs showed biophysical characteristics similar to serum-derived VLDL, indicating a functional lipoprotein metabolism. We could confirm expression of HCV entry factors in mature iHLCs as well as permissiveness to cell culture–derived HCV. RNA replication and particle production were supported after the differentiating cells reached the stage of immature hepatocytes. Further, several interferon-stimulated genes (ISGs) were induced upon HCV infection in iHLCs, an effect that was not observed in Huh7 and Huh7.5 cells, despite a higher viral load. In contrast, interferon-α-stimulation induced ISG expression in all cell types, suggesting that pathogen recognition is intact in iHLCs and diminished in the hepatoma cells. Blocking JAK-STAT-signalling increased viral replication in mature iHLCs, together with an abolished induction of ISGs. Additionally, we analysed HCV replication in iHLCs with shRNA-mediated downregulation of certain parts of the antiviral signalling cascade.

## Results

### iPSCs successfully differentiate into iHLCs

**Figure 1:**
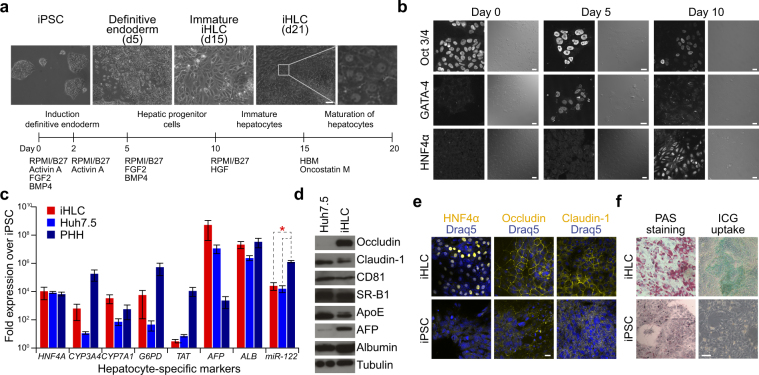
Differentiation and characteristics of iHLCs. (**a**) Cell morphology and culture conditions at different stages of differentiation from iPSCs into iHLCs. Bright-field images were taken at the indicated time points (scale bar 100 µm). (**b**) Sequential expression and repression of transcription factors marking successful differentiation. Cells were immunostained with antibodies against Oct 3/4 (pluripotency marker), the endodermal transcription factor GATA-4, and the hepatic marker HNF4α at day 0, 5, and 10 of differentiation (scale bars 20 µm). Shown are representative fluorescence and DIC images. (**c**) mRNA expression levels of different hepatocyte-specific factors in iHLCs, Huh7.5 cells, and primary human hepatocytes (PHHs) were analysed via qRT-PCR. Expression levels are displayed as fold over iPSCs normalised to *GAPDH* and *18S rRNA* (mean ± SEM, n = 3–4, *p < 0.05). (**d**) Western blot analysis of protein expression in HCV-infected iHLCs compared to Huh7.5 cells of different host factors crucial for HCV infection. Tubulin served as loading control. Full-length blots are presented in Supplementary Figure 1. (**e**) iHLCs and iPSCs were immunostained for the hepatic marker HNF4α and the tight junction proteins occludin and claudin-1 (nuclei were visualised with Draq5) (scale bar 20 µm). (**f**) Metabolic functionality of mature iHLCs was evaluated by analysing glycogen storage by Periodic acid-Schiff (PAS) staining and indocyanine green (ICG) uptake visualised by bright-field microscopy (scale bar 100 µm). Shown are representative bright-field images.

We first assessed the successful differentiation from iPSCs into iHLCs. Changes in cell morphology together with the sequential repression and expression of different lineage- and tissue-specific markers confirmed the progression through differentiation at several stages (Fig. 1a and b). The pluripotency marker Oct 3/4 was detectable by immunofluorescence staining in naïve iPSC colonies (d0) and less prominent at day 5 and 10. Induction of the definitive endoderm was indicated by GATA-4 expression at day 5, which was repressed again at day 10. Differentiation towards a hepatic fate was marked by the continuous expression of the hepatic transcription factor HNF4α after day 10. Mature iHLCs displayed cobblestone morphology and sometimes were even binuclear (Fig. 1a). Further, mature iHLCs expressed hepatocyte-specific enzymes like the cytochrome p450 isoforms cholesterol 7-alpha-monooxygenase (*CYP7A1*) and cytochrome P450 3A4 (*CYP3A4*), the glucose-6-phosphate 1-dehydrogenase (*G6PD*) and the tyrosine aminotransferase (*TAT*) as well as albumin (*ALB*), α-fetoprotein (*AFP*) and the transcription factor HNF4α (Fig. 1c). However, mRNA expression levels of the respective genes were more similar to expression in the hepatoma cell line Huh7.5 than to primary human hepatocytes (PHHs). The rather high expression of AFP indicates incomplete maturation, a phenotype previously described for iHLCs^15,25,29,30^.

### iHLCs are metabolically functional and express HCV host factors

Since we are interested in establishing iHLCs as an alternative model to study HCV-host interactions, we next determined the protein expression of HCV host factors in mature iHLCs via western blot analysis and compared them to the hepatoma cell line Huh7.5, a derivate of the Huh7 cell line that is even more permissive for HCV and the most commonly used cell line to study HCV *in vitro*. The entry factors scavenger receptor B1 (SR-B1), CD81, and claudin-1 showed equivalent protein levels, whereas occludin was expressed to an even higher extent (Fig. 1d). Expression of the tight junction proteins claudin-1 and occludin as well as the hepatocyte marker HNF4α was additionally visualised by immunofluorescence staining. In contrast to iPSCs, mature iHLCs displayed a distinct localisation of the tight junction proteins at the cell membrane and nuclear expression of HNF4α (Fig. 1e). Protein expression of ApoE, an important host protein for HCV particle production, and the hepatocyte marker albumin was equal in Huh7.5 and iHLCs (Fig. 1d). Another liver-specific host factor required for HCV replication, miR-122^31^, was also expressed equally compared to Huh7.5 cells, but less than in PHHs (Fig. 1c). To sum up, iHLCs express HCV host factors known to be required for successful infection and replication *in vitro*. Next, we addressed if iHLCs exhibit characteristics of liver metabolic functions. Together with the skeletal muscle, liver cells are the major site of glycogen storage. Periodic acid-Schiff (PAS) staining of iHLCs and iPSCs revealed a high amount of glycogen stored in mature iHLCs (Fig. 1f). Further, we treated the cells with indocyanine green (ICG) in an assay to monitor hepatic function. In contrast to naïve iPSCs, we observed green colonies of iHLCs, indicating a functional metabolic uptake (Fig. 1f).

### iHLCs secrete VLDL-like particles

**Figure 2:**
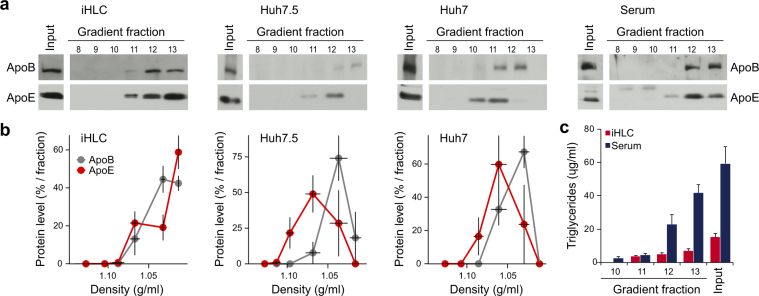
Lipoprotein secretion profile of iHLCs and hepatoma cells. (**a**) Western blot analysis of density gradient fractions with antibodies against ApoE and ApoB. For characterisation of lipoproteins secreted by iHLCs, Huh7, and Huh7.5 cells, cell culture supernatants were subjected to linear iodixanol density gradients. For comparison of lipoprotein distribution, human serum was analysed as well. Shown is one representative experiment. Full-length blots are presented in Supplementary Figures 2 and 3. (**b**) Distribution of ApoE and ApoB within the gradient. Western blot signals for ApoE and ApoB were quantified using the densitometric quantification function of Fiji. Shown is the relative amount of lipoproteins as per cent of total plotted against the density of the respective fraction (n = 3). All full-length blots used for quantification are presented in Supplementary Figures 2 and 3. (**c**) Determination of triglycerides in gradient fractions of iHLCs and human serum (mean ± SEM, n = 3).

HCV is known to interact with the hepatic lipoprotein metabolism to mature to LVPs^5^. The majority of viral RNA from serum-derived samples can be isolated from triglyceride-rich particles, containing ApoE and ApoB, at a density of ≤1.08 g/ml^32,33^, suggesting that HCV associates with very-low density lipoproteins (VLDLs) to form LVPs. Huh7 cells and their derivates have an impaired VLDL metabolism and as a result secrete HCV particles with a higher density and lower specific infectivity compared to primary human hepatocytes^3,4^. To assess the biophysical properties of lipoproteins secreted by iHLCs, we performed density gradient centrifugation. Supernatants of mature iHLCs and concentrated supernatants of Huh7 and Huh7.5 cells were layered on top of linear iodixanol gradients, subjected to ultracentrifugation, and fractions were harvested from the bottom. Human serum was analysed as a control for *bona fide* lipoproteins. First, we determined the amount of ApoE and ApoB in the gradient fractions by western blotting and measured the respective densities (Fig. 2a). In gradients of the iHLC supernatant both apolipoproteins, ApoE and ApoB, were detectable in the top fractions, correlating with a density range of 1.067–1.0136 g/ml. A similar distribution was observed within the human serum gradient. In contrast, ApoE in gradients of Huh7 and Huh7.5 supernatants was shifted to a higher density. ApoE and ApoB in gradients of hepatoma cells were detectable within a density range of 1.0856–1.0298 g/ml for Huh7 and 1.0898–1.0272 g/ml for Huh7.5 cells, respectively. Interestingly, ApoE from the Huh7 and Huh7.5 supernatants peaked at a higher density than the ApoB (Fig. 2b). In contrast, maxima for both apolipoproteins secreted by iHLCs were found in the same fractions of the lowest density. This joint distribution was also observed for apolipoproteins from the human serum (Fig. 2a). As VLDLs are also characterised by their high triglyceride content, we determined the amount of triglycerides in the apolipoprotein containing fractions. Here, we detected the majority of triglycerides in the upper fractions of the serum- and the iHLC gradients. Highest concentrations were observed in fractions with the lowest density showing a continuous decrease while density increases. Due to technical issues, related to interference of concentrated OptiMEM with the triglyceride assay, we were not able to determine the triglycerides in the hepatoma cell gradient fractions.

### iHLCs get permissive to HCV infection during differentiation

**Figure 3:**
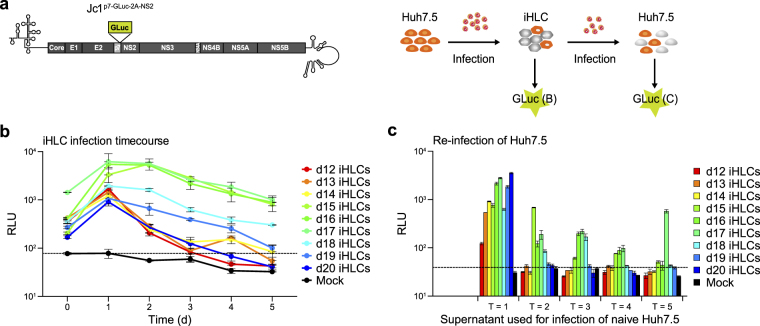
Immature and mature iHLCs are permissive to HCV infection. (**a**) Scheme of the experimental setup. (**b**) Permissivity at different stages of differentiation. Cells were infected with cell culture derived Jc1^p7-GLuc-2A-NS2^ from day 12–20 of differentiation and viral replication was monitored for 7 days. 24 h post infection, cells were washed and the last wash was kept as T = 0. Gaussia luciferase activity was measured in the supernatant and in the washes after media change at the indicated time points. The dashed horizontal line represents the background luciferase activity determined in mock-infected iHLCs. (**c**) Re-infection of Huh7.5 cells with supernatant collected during (**b**). Huh7.5 cells were infected with the iHLC supernatant from T = 1–5 to determine the production of infectious viral particles at different stages of differentiation. Gaussia luciferase activity was measured 2 days post re-infection (Mean ± SD, duplicate measurements). The dashed horizontal line represents the background luciferase activity determined in mock-infected Huh7.5 cells.

To further evaluate our model as functional for HCV *in vitro* studies, we analysed permissiveness to HCV infection and production of infectious viral particles. First, we determined the stage of differentiation in which the cells become permissive to HCV. For monitoring HCV replication, we used a Jc1 reporter strain encoding for a secreted gaussia luciferase (GLuc) followed by a modified F2A ribosomal skipping site between p7 and the NS2 protein (Jc1^p7-GLuc-2A-NS2^)^34^ (Fig. 3a). Cells were infected with Jc1^p7-GLuc-2A-NS2^ viral stocks at different time points (day 12–20) of differentiation, washed at 24 h post infection and the last wash was kept as T = 0. We measured luciferase activity as a proxy for viral replication at the indicated time points (Fig. 3b). For all infections, viral replication increased from T = 0 to T = 1. Highest overall replication rates were observed for cells infected at the beginning of maturation (day 15–17). For infections on day 15–18 of differentiation, viral replication was steady or even increasing (day 15) until T = 2. In all infections, we observed a decrease in viral replication over time independent of the differentiation stage at the day of infection. In order to evaluate the production of infectious viral particles by the differentiating cells, we used the supernatant collected at the different time points to re-infect naïve Huh7.5 cells and measured luciferase activity 2 days post infection (Fig. 3c). Highest replication rates were measured for Huh7.5 cells infected with the supernatant of T = 1 of the individual infections. Only when the differentiating cells were infected between days 15–18 we were able to recover infectious particles over time. Both earlier and later infection lead to a fast loss of viral particle production.

### HCV infection induces interferon-stimulated genes in iHLCs

**Figure 4:**
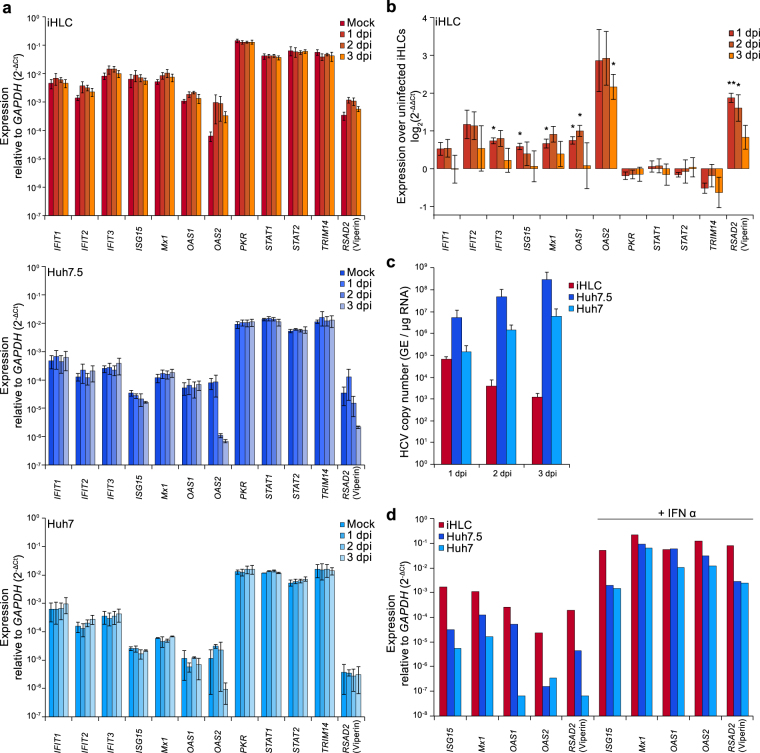
Induction of ISG expression after HCV infection is stronger in iHLCs compared to hepatoma cells. (**a**) mRNA expression levels of interferon stimulated genes (ISGs) in iHLCs, Huh7.5, and Huh7 cells were determined by qRT-PCR at day 1, 2, and 3 days post infection (dpi) with cell culture–derived HCV (fresh concentrate of Jc1/JFH1 for iHLCs; Jc1 (MOI = 0.01) for Huh7 and Huh7.5). Shown are expression levels relative to *GAPDH* (mean ± SEM, n = 2–3). (**b**) Induction of ISGs in iHLCs after HCV infection as log_2_-fold over uninfected control (normalised to *GAPDH*, mean ± SEM, n = 3, *p < 0.05, **p < 0.01) (**c**) HCV RNA copy number was measured by qRT-PCR at the indicated time points after infection. Copy number per total µg RNA was calculated using a serial dilution of cDNA derived from *in vitro* transcribed HCV RNA (mean ± SEM, n = 2–3). (**d**) ISG mRNA expression levels after 24 h treatment with 1000 U/ml recombinant IFNα were determined by qRT-PCR. Shown is one representative experiment.

As the hepatoma cell lines Huh7 and Huh7.5 cells are known to be deficient in antiviral signalling^8,10^, we assessed if iHLCs display a more intact innate immunity and therefore are of better use to study virus-host interactions. Here, we analysed the induction of several ISGs in context of HCV infection. As primary human hepatocytes are possibly more permissive to the non-adapted JFH1, but Jc1 reaches high titers in cell culture^35^, iHLCs were infected with a mixture of Jc1^wt^ and JFH1^wt^ viral stocks to increase infection rates, while Huh7 and Huh7.5 cells were infected with Jc1^wt^. Total RNA was isolated at day 1–3 post infection and ISG expression levels as well as HCV RNA copy numbers were determined by qRT-PCR (Fig. 4a–c). We compared the expression of twelve ISGs that have been described previously to be induced upon HCV infection in primary human hepatocytes or iHLCs, or by IFNλ-stimulation in iHLCs or Huh7 cells^26,36–39^. mRNA expression levels were determined by qRT-PCR in uninfected cells and at day 1–3 post infection (Fig. 4a). To illustrate the differences in basal expression levels the values are shown relative to *GAPDH*, expressed as 2^−ΔCt^. Basal expression of almost all ISGs was approximately 10-fold higher in iHLCs as compared to the hepatoma cells. In addition, almost no induction of ISGs was observed in the HCV-infected hepatoma cell lines. In these experiments Huh7/7.5 cells were infected with a multiplicity of infection (MOI) of 0.01. Of note, even infection with a 100-fold higher MOI of 1 did not induce ISGs (data not shown). In contrast, we observed a modest induction of the 2′-5′-oligoadenylate synthetase 2 (*OAS2*) as well as radical S-adenosyl methionine domain containing 2 (*RSAD2* or Viperin) in iHLCs in response to HCV infection (Fig. 4a and b). A slight induction by HCV was additionally observed for the interferon induced protein with tetratricopeptide repeats (*IFIT*) 1, 2, and 3, as well as *ISG15*, *Mx1*, and *OAS1* (Fig. 4b). In contrast, the basally highly expressed *PKR*, *STAT1*, *STAT2*, and *TRIM14* did not respond to HCV infection in iHLCs. Overall, basally low expressed genes showed a higher induction by HCV infection than genes that were already highly expressed in uninfected cells. Intriguingly, when we analysed HCV RNA copy numbers in the same samples, we observed a concomitant decline in iHLCs while in the hepatoma cell lines copy numbers steadily increased (Fig. 4c). As a control, we additionally stimulated all cell types with recombinant interferon alpha (IFNα) and compared the mRNA expression levels of *ISG15*, *Mx1*, *OAS1*, *OAS2*, and *RSAD2* to untreated cells. Here, we observed a strong induction of all analysed ISGs in iHLCs but also in the hepatoma cell lines, demonstrating an intact interferon response (Fig. 4d). Taken together, iHLCs show a stronger induction of ISGs in presence of a lower viral load than Huh7 and Huh7.5 cells, indicating a functional antiviral response.

### HCV replication is increased in iHLCs by inhibition of JAK-STAT-signalling

**Figure 5:**
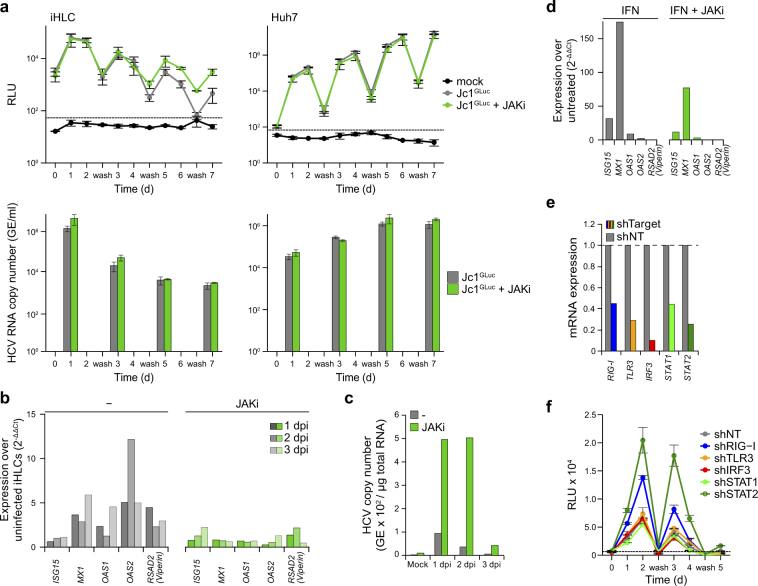
Inhibition of antiviral signalling in iHLCs increases HCV replication. (**a**) HCV replication in presence of JAK-STAT pathway inhibition. iHLCs or Huh7 cells were pre-treated with 10 µM InSolution JAK inhibitor (JAKi) for 1 h and infected with Jc1^p7-GLuc-2A-NS2^. iHLCs were infected at day 16 of differentiation (immature hepatocytes). 24 h post infection, cells were washed (T = 0) and gaussia luciferase activity in the supernatant was monitored to quantitate viral replication. JAKi treatment and media were renewed daily (mean ± SEM, n = 2–3). Additionally, viral RNA was isolated from the cell supernatant at the indicated time points and the HCV RNA copy number was measured by qRT-PCR. (**b**) ISG mRNA expression levels in iHLCs with and without JAKi-treatment were determined by qRT-PCR. Cells were pre-treated with JAKi 1 h prior to infection with cell culture–derived Jc1/JFH1 or left untreated. Medium was changed 3 h post infection. Total RNA was isolated at 1, 2, and 3 dpi. Shown are log_2_-fold changes over uninfected cells (normalised to *GAPDH*). (**c**) HCV RNA copy number from (**b**) was measured by qRT-PCR at the indicated time points after infection. Copy number per µg total RNA was calculated using a serial dilution of cDNA derived from *in vitro* transcribed HCV RNA. (**d**) ISG mRNA expression of Huh7 cells treated with IFNα with and without prior JAKi-treatment. Shown is one single representative experiment. (**e,f**) Immature iHLCs were transduced with lentiviral particles carrying shRNAs against RIG-I, TLR3, IRF3, STAT1, and STAT2, or non-targeting (NT) control at day 12 and day 13 of differentiation. At day 19, cells were infected with Jc1^p7-GLuc-2A-NS2^. Knockdown of the respective target gene was verified by qRT-PCR (**e**) and viral replication was determined by measurement of gaussia luciferase activity in the supernatants (**f**) (Mean ± SD, duplicate measurements).

Based on previous results by others and on our observation of ISG induction and concomitant decrease of HCV copy numbers in iHLCs, we analysed HCV replication in presence of JAK-STAT pathway inhibition. As iHLCs were permissive to HCV infection from day 15 of differentiation onwards (Fig. 3b), we assessed the influence of JAK-STAT inhibition in cells infected at day 16 of differentiation. Cells were treated with a small molecule inhibitor of JAK (JAKi) 1 h prior to infection with the HCV reporter Jc1^p7-GLuc-2A-NS2^. HCV replication was monitored by gaussia luciferase activity assays for 7 days with media changes and washes every other day. HCV replication rates in JAKi-treated cells were similar as in control cells in the early stages of maturation (T = 1–4, corresponding to 2–5 dpi, day 18–21 of differentiation) (Fig. 5a). However, in mature iHLCs, viral replication rates were higher and more stable in the presence of JAK-STAT inhibition (T = 5–7, corresponding to 6–8 dpi, day 22–24 of differentiation). In contrast, treatment of Huh7 cells with JAKi prior to and during infection did not change HCV replication rates (Fig. 5a). Additionally, we determined the HCV RNA copy numbers in the supernatant at the indicated time points to follow changes in viral titres over time (Fig. 5a). Here, we observed slightly higher HCV RNA copy numbers in supernatants of JAKi-treated iHLCs at early time points (T1 and T3), while later time points showed similar HCV RNA levels. In line with the luciferase assays for replication, viral RNA copy numbers decreased over time. In contrast, we observed a steady and similar increase of HCV RNA copy numbers in the supernatant of JAKi treated and untreated Huh7 cells. Next, we addressed if the induction of ISGs is blocked by JAK-STAT inhibition. iHLCs were infected with a mixture of Jc1^wt^ and JFH1^wt^ in presence or absence of JAKi and total RNA was isolated at day 1–3 post infection. As expected, JAKi treatment counteracted the induction of ISGs upon HCV infection (Fig. 5b). HCV RNA copy numbers were already slightly higher in cells treated with JAKi at these early time-points after infection, consistent with the higher levels in the supernatant (Fig. 5c). To confirm that the JAKi can block ISG induction in Huh7 cells, we treated them with interferon α and observed that indeed JAKi-treatment counteracts interferon-induced induction of all ISGs tested (Fig. 5d). To further dissect the blockade of the antiviral response and its role in HCV replication in iHLCs, we knocked down different factors of the innate immunity using shRNAs and infected the cells with the HCV reporter Jc1^p7-GLuc-2A-NS2^. Cells were transduced on two consecutive days (day 12 and day 13 of differentiation) with lentiviral particles encoding shRNAs targeting RIG-I, TLR3, STAT1, STAT2, IRF3, or a non-targeting (NT) control. After maturation, iHLCs were infected with Jc1^p7-GLuc-2A-NS2^ and HCV replication was measured for 5 days. At the end of the experiment, we isolated RNA and confirmed the knockdown induced by the shRNAs by qRT-PCR (Fig. 5e). RIG-I knockdown slightly and STAT2 knockdown strongly increased HCV replication compared to the control cells (Fig. 5f). In contrast, knockdown of TLR3, STAT1, or IRF3 did not change viral replication compared to the control. Thus sensing of incoming viral RNA by RIG-I and induction of an antiviral state via STAT2 suppresses HCV replication in iHLCs.

## Discussion

In the present study we sought to evaluate iPSC-derived iHLCs as a model for studying HCV host interactions *in vitro*. Using an established protocol^14^, iPSCs successfully differentiated into hepatocyte-like cells. However, mRNA expression levels of hepatic markers were more similar to Huh7.5 cells than to adult PHHs. The higher expression level of AFP suggests that iHLCs are not terminally differentiated but resemble foetal hepatocytes, a phenotype that has been observed by others^15,29,30^. iHLCs expressed the main HCV entry receptors as well as important host factors for viral replication and virion production, such as miR-122 and ApoE, which is in line with other reports and differentiation procedures^23–26^. Importantly, iHLCs secreted ApoE that co-fractionated with ApoB in density gradient centrifugations in the fraction with the lowest density. This indicates that iHLCs have the capability to secret *bona fide* VLDLs. In contrast, Huh7-derived cell lines secreted ApoE and ApoB independently with ApoE peaking at a higher density than ApoB. Therefore, our results confirm defective VLDL assembly and secretion in Huh7-derived cells observed by others^3,4^. This defect is described to prevent the fusion of the HCV precursor with the ApoB-containing VLDL-precursor^40^. Accordingly, and in contrast to HCV particles from patient sera or PHHs, cell culture–derived HCV particles have a lower density and are mainly associated with ApoE^6,41^.

iHLCs were permissive to HCVcc infection, supported the full viral life cycle, and produced infectious viral particles. In an infectivity time course, HCV replication was supported after cells reached the stage of immature hepatocytes. This is in line with recent results that hepatic specification of the cells marked the transition point for permissiveness and hepatic maturation was not required for successful infection^23^. In our experiments, we observed a higher and more robust HCV replication, along with the production of infectious viral particles, in cells infected at day 15–17 of differentiation as compared to cells that were infected at later time points of maturation. Still, following the initial infection, HCV was cleared in iHLCs after 1–5 days. Exposure of PHHs or stem cell–derived hepatocytes to HCV resulted in induction of different ISGs and inflammatory chemokines^25,27,28,37,38^, correlating with a drop in the viral load^28,38,39^. To this end, we compared the expression of twelve genes either linked to the induction of innate immunity (*STAT1*, *STAT2*, *PKR*) or IFN effector genes (*OAS1/2*, *IFIT1–3*, *RSAD2*, *MX1*, *TRIM14*, *ISG15*) in HCV-infected hepatoma cells and mature iHLCs. We observed a modest but significant upregulation of *IFIT3*, *ISG15*, *MX1*, *OAS1, OAS2*, and *RSAD2* (Viperin) in infected iHLCs that was mostly not present in Huh7 cell lines. In contrast, we did not observe an induction of *TRIM14*, *STAT1*, *STAT2*, and *PKR*; these genes already showed a high basal expression in uninfected cells compared to the other ISGs and HCV infection did not further increase mRNA levels. Of note, even though Huh7.5 cells harbour a dominant-negative RIG-I allele and the parental Huh7 cells express functional RIG-I^8^, both cell lines respond less to HCV infection than iHLCs, despite higher viral loads, and independent of the MOI (up to 1) used for initial infection. One explanation is that the fast infection kinetics in hepatoma cells allows HCV to counteract the innate response more effectively. Laser-dissection followed by microarray analysis of HCV-infected PHHs revealed that a number of ISGs was significantly upregulated the infected- as well as the adjacent cells at early time points post infection^38^, indicating that the ISG induction we observed might reduce HCV infection in two ways: First, directly counteracting viral replication in the infected cells and second limiting cell-to-cell spread by inducing an antiviral state in surrounding cells. Future studies have to dissect which of the upregulated ISGs are dominant in limiting viral spreading in iHLCs. Interestingly, ISGs were inducible upon IFNα treatment in all cell types, suggesting that the sensing of viral pathogen-associated molecular patterns (PAMPs) rather than the signalling cascade is disrupted in hepatoma cells^8,10,28,36^.

Recognition of pathogens by the innate immune system induces an interferon response via the JAK-STAT signalling pathway^42^. Treatment of iHLCs with a JAK inhibitor increased viral replication while no changes were observed in Huh7 cells, supporting the hypothesis that HCV infection in iHLCs is lower because of an intact innate immunity^26,37^. We were unsuccessful to directly measure interferon induction in our system likely due to the low levels of infection in iHLCs. However, we observed a reduced induction of ISGs in JAKi-treated iHLCs, indicating an interferon-mediated induction. HCV replication in JAKi-treated cells did not increase until iHLCs fully matured, implying a more efficient antiviral response in mature than in immature cells^26,27^. This result is underpinned by our observation that immature cells infected at day 15–17 display a higher HCV replication and produce more infectious particles. JAKi-treatment of iHLCs also led to slightly higher intra- and extracellular HCV RNA copy numbers at early time points post infection. Therefore blunting JAK-STAT signalling promotes initial infection of iHLCs, an effect that might lead to a higher proportion of infected cells (initial or by spreading) that maintain replication for a longer period of time. We also observed increasing replication in cells with downregulated *STAT2* expression. In contrast, the knockdown of *STAT1* did not affect HCV replication. Recently, it has been reported that STAT1, but not STAT2, is dispensable for IFNα-induced inhibition of HCV replication, while both are required for IFNλ-signalling^43^. Therefore, our data suggests that in iHLCs, HCV replication is, at least partly, controlled by IFNα-induced antiviral signalling. Nonetheless, there is emerging evidence that IFNλ plays an important role in HCV infection^37,38,44^.

Upon infection, viral genomes are sensed by different pattern recognition receptors (PRRs)^45^. RIG-I and TLR3 were described to activate antiviral signalling upon HCV infection^8,9,46–48^. Here, we observed that disruption of viral sensing by downregulation of *RIG-I* increased viral replication, whereas the knockdown of *TLR3* had no effect. Of note, expression of *TLR3* was relatively low and its role might be compensated by RIG-I. In addition, TLR3 is only activated by the dsRNA HCV replication intermediates^46^, which might be in low number, given the low viral load detected in the iHLCs. Another member of the RIG-I-like receptor family that we did not analyse in this work, the RNA helicase MDA5, has recently been described to sense HCV in infected cells and to activate the interferon response^49^. Future experiments should address the role of MDA5 in the innate immune response in iHLCs. Downregulation of *IRF3*, a key transcription factor in type I IFN and ISG response and a downstream molecule of PRR signalling^50^, also did not change replication levels. One possible explanation for this observation is that the transcription factors NFκB and IRF7, which are also downstream of the RIG-I-MAVS signalling cascade, can balance the loss of IRF3^51^. This supports previous reports that multiple ways of antiviral signalling work together in controlling HCV infection^52^. Taken together, our data shows that iHLCs display a functional innate immunity upon HCV infection that contributes to a decrease in viral replication.

In conclusion, our data confirms that iHLCs provide an HCV *in vitro* model with a higher physiological relevance than the currently used hepatoma cells. Like others, we detected an intact innate immunity in iHLCs, allowing the analysis of virus-host interactions in antiviral immunity. Given that spontaneous viral clearance is, at least partly, dependent on host genetics and the pre-infectious level of ISGs, iHLCs provide a suitable model to study host determinants in a broader range^53^. With regard to the intact VLDL synthesis, our iHLCs provide a platform for the possible formation of HCV lipoviroparticles with *in vivo* characteristics. Further, studying the behaviour of different HCV genotypes as well as clinical isolates in hepatoma cell lines is still difficult^54–57^. Recently published data shows that stem cell-derived HLCs are more susceptible to patient isolates than Huh7.5 cells^23^, supporting the conclusion that the usage of iHLCs might overcome this problem and broadens the possibilities of studying HCV host interaction *in vitro*.

## Materials and Methods

### Cell lines and culture conditions

Huh7.5 cells from C. Rice, Huh7 cells from R. Bartenschlager and HEK293T cells obtained from the American Type Culture Collection Culture were cultured in high glucose DMEM (Life Technologies) supplemented with 10% FCS (Biochrom), 1% GlutaMAX. The iPSC clone #179 (HEXT stem cell core facility, University Clinic Hamburg Eppendorf) was cultured in Matrigel (BD Bioscience)-coated 6-well plates in MEF-conditioned medium (MEF-CM; DMEM/F12 Ham, 5% KO serum replacement, 1 mM L-glutamine, 9.8 µM β-mercaptoethanol, non-essential amino acids, P/S, 4 ng/ml FGF2), supplemented with 4 ng/ml FGF2. Medium was renewed daily; cells were split using EDTA and resuspended in MEF-CM supplemented with 10 µg/ml ROCK-Inhibitor (Y-27632 dihydrochlorid, Cayman).

Differentiation of iPSCs into iHLCs was performed as described before^14^. 24 h prior to differentiation, cells were seeded to Matrigel-coated 6- or 12-well cell culture plates to reach ~90% confluency. To induce definitive endoderm formation, cells were cultured for two days in RPMI/B27-Insulin (RPMI 1640, 1% GlutaMAX, 2% B27-Ins, 0.5% non-essential amino acids, 0.5% Penicillin/Streptomycin) containing 100 ng/ml activin A (R&D Systems), 10 ng/ml BMP4 (Life Technologies) and 20 ng/ml FGF2 (PeproTech), followed by three days in RPMI 1640/2% B27-Ins supplemented with 100 ng/ml activin A only. At day 6, medium was changed to RPMI 1640/2% B27 + Ins containing 20 ng/ml BMP4 and 10 ng/ml FGF2. For hepatic differentiation, cells were cultured for five days in RPMI 1640/2% B27 + Ins supplemented with 20 ng/ml HGF (PeproTech), followed by five days cultivation in HBM culture medium (HCM BulletKit without adding EGF; Lonza) containing 20 ng/ml oncostatin M (PeproTech). The medium was changed daily.

### Density gradient centrifugation for lipoprotein analysis

iHLC cell culture supernatant was collected from cells differentiated in 6-well plates and directly used for analysis. For Huh7/7.5 cells ~1.5 × 10^6^ cells were cultured in 12 ml serum free medium (OptiMEM) overnight. The supernatant was concentrated to ~1.5 ml using centrifugal filter units (Amicon Ultra 15 ml, MWCO 100 kDa, Millipore). Linear density gradients were prepared as described before with 6% (wt/vol) (1.7 ml of 60% iodixanol, 0.34 ml of 0.5 M Tris/HCl, pH 8.0, 0.34 ml of 0.1 M EDTA, pH 8.0, and 14.6 ml 0.25 M sucrose) and 56.4% (wt/vol) (16.0 ml of 60% iodixanol, 0.34 ml of 0.5 M Tris/HCl, pH 8.0, 0.34 ml of 0.1 M EDTA, pH 8.0, and 0.34 ml 0.25 M sucrose) iodixanol solutions^32^. ~1 ml of iHLC or concentrated Huh7/7.5 cell culture supernatant was layered on top and ultracentrifugation was performed in an SW41 rotor (Beckman Coulter) at 207,570 × *g*, 4 °C for 16 h. 1 ml fractions were harvested from the bottom. Proteins from hepatoma cell input and gradient fractions were precipitated using trichloroacetic acid/acetone^58^. Briefly, 600–800 µl per fraction were mixed with 15% trichloroacetic acid and 30% acetone and incubated for 30 minutes at 4 °C. Prior to precipitation, 100 µg BSA was added as carrier protein. After centrifugation at 15,000 × *g* for 20 minutes at 4 °C, protein pellets were washed with 1 ml 100% acetone, air dried, and resuspended in 100 µl urea loading dye (8 M urea, 200 mM Tris/HCl pH 6.8, 1 mM EDTA, 15 mM DTT, 5% SDS, 0.1% bromophenol blue).

Density gradient fractions were analysed for lipoprotein distribution by western blotting using antibodies directed against ApoE and ApoB. Signal intensities of the respective bands were determined using the densitometric function of Fiji.

The amount of triglycerides in iHLC and serum gradient fractions was determined using the Infinity triglycerides reagent (Thermo Fisher Scientific). Briefly, 150 µl of reagent was added to 10–30 µl of the sample, incubated for 1 h at 37 °C, and analysed with a plate reader (Infinite M200, Tecan).

### Western blot analysis

Cells were lysed in RIPA lysis buffer (150 mM NaCl, 50 mM Tris/HCl pH 7.6, 1% Nonidet P40, 0.5% sodium deoxycholate, 5 mM EDTA supplemented with 1 × protease inhibitor cocktail (Sigma)) for 30–60 minutes on ice. For western blotting, equal amounts of protein or equal volumes of gradient fraction samples were subjected to SDS-PAGE. Signals were detected by chemiluminescence using Lumi-Light (Roche) or SuperSignal West Femto (Thermo) western blotting substrate on ECL Hyperfilm (Amersham).

### RNA Isolation and quantitative RT-PCR

Total cellular RNA was isolated using Trizol (Sigma or Tel-Test). Viral RNA from cell culture supernatants was isolated using the Nucleo Spin RNA Virus Kit (Macherey-Nagel). After DNAse treatment (DNA-free, Life Technologies), equal amounts of RNA were reverse transcribed using SuperScript III (Life Technologies) with random hexamer primers (Qiagen). For cDNA synthesis of miR-122, we used a specific RT-Primer (GTCGTATCCAGTGCGTGTCGTGGAGTCGGCAATTGCACTGGATACGACCAAACAC). Quantitative real time PCR was performed using Maxima SYBR Green (Life Technologies) and the primers listed below on a 7500 Fast Real Time PCR System (Applied Biosystems).

Primers selected from the PrimerBank^59^ were used for qPCR (AFP fw, GCAGCCAAAGTGAAGAGG; AFP rev, TGTTGCTGCCTTTGTTTG; ALB fw, GGCACAATGAAGTGGGTAAC; ALB rev, AGGCAATCAACACCAAGG; CYP3A4 fw, CCTTACATATACACACCCTTTG; CYP3A4 rev, GGTTGAAGAAGTCCTCCTAAGCT; CYP7A1 fw, CTGCCAATCCTCTTGAGTTCC; CYP7A1 rev, ACTCGGTAGCAGAAAGAATACATC; G6PD fw, TTCCCTGTAACCTGTGAGAC; G6PD rev, ATTCAAGCACCGAAATCTG; GAPDH fw, AAGGTGAAGGTCGGAGTCAAC; GAPDH rev, GGGGTCATTGATGGCAACAATA; IRF3 fw, AGAGGCTCGTGATGGTCAAG; IRF3 rev, AGGTCCACAGTATTCTCCAGG; ISG15 fw, TGGACAAATGCGACGAACCTC; ISG15 rev, TCAGCCGTACCTCGTAGGTG; IFIT1fw, GCGCTGGGTATGCGATCTC; IFIT1rev, CAGCCTGCCTTAGGGGAAG; IFIT2 fw, GACACGGTTAAAGTGTGGAGG; IFIT2 rev, TCCAGACGGTAGCTTGCTATT; IFIT3 fw, AGAAAAGGTGACCTAGACAAAGC; IFIT3 rev, CCTTGTAGCAGCACCCAATCT; miR122 fw, GGGGTGGAGTGTGACAATG; miR122 rev, CAGTGCGTGTCGTGGAGT; MX1 fw, GGTGGTCCCCAGTAATGTGG; MX1 rev, CGTCAAGATTCCGATGGTCCT; OAS1 fw, AGCTTCGTACTGAGTTCGCTC; OAS1 rev, CCAGTCAACTGACCCAGGG; OAS2 fw, CTCAGAAGCTGGGTTGGTTTAT; OAS2 rev, ACCATCTCGTCGATCAGTGTC; PKR fw, GCCGCTAAACTTGCATATCTTCA; PKR rev, TCACACGTAGTAGCAAAAGAACC; TAT fw, ACTGTGTTTGGAAACCTGCC; TAT rev, GCAGCCACTTGTCAGAATGA; TLR3 fw, TTGCCTTGTATCTACTTTTGGGG; TLR3 rev, TCAACACTGTTATGTTTGTGGGT; TRIM14 fw, TACATTACAGACGCCATTGGAC; TRIM14 rev, GGGCTGGTTTTCAACAAGGT; STAT1 fw, CGGCTGAATTTCGGCACCT; STAT1 rev, CAGTAACGATGAGAGGACCCT; STAT2 fw, CTGCTAGGCCGATTAACTACCC; STAT2 rev, TCTGATGCAGGCTTTTTGCTG; RIG-I fw, TGTGCTCCTACAGGTTGTGGA; RIG-I rev, CACTGGGATCTGATTCGCAAAA; RSAD2 (Viperin) fw, TTGGACATTCTCGCTATCTCCT; RSAD2 (Viperin) rev, AGTGCTTTGATCTGTTCCGTC) and HCV-specific primers as described^58^ (JFH1 fw, CGGGAGAGCCATAGTGG; JFH1 rev, AGTACCACAAGGCCTTTCG).

### HCV and lentiviral plasmids

For HCV infections we used wild type JFH1 (subcloned into pBR322) and Jc1 constructs as described^35,60^. The HCV reporter Jc1^p7-GLuc-2A-NS2^ was cloned as described^34^, but with a modified F2A site. For shRNA constructs, the primers listed below were cloned into a modified pSicoR-MS1-Puro vector^61^ using the following target sequences: IRF3, GGAGGCAGTACTTCTGATA; RIG-I, GAAACTTGCCAGTTATATA, STAT1, GGTGGTATTTAGTCTATTA; STAT2, GAACAGTCCTGTTCAGAAA; TLR3, GGAGATTCCAGATTATAAA, and NT-control, GTCAAGTCTCACTTGCGTC described in^62^. Complete primer sequences are available on request.

### Immunofluorescence and microscopy staining

Immunofluorescence staining for confocal microscopy was essentially performed as described before^61^. Briefly, iPSCs were seeded on Matrigel-coated coverslips for differentiation and fixed with 4% paraformaldehyde. After permeabilisation with 0.1% Triton X-100 for 5 minutes, cells were incubated in blocking solution (5% BSA, 1% fish skin gelatine, 50 mM Tris in PBS) followed by staining with primary (diluted in blocking solution, 1 h at RT or o/n at 4 °C) and secondary (1 h at RT) antibodies and embedding in Mowiol. Images were acquired using a Zeiss LSM510 confocal microscope equipped with a Plan-Neofluar 40×/1.3 oil objective. For Periodic acid Schiff (PAS) staining, we used the PAS staining kit (Carl Roth) and indocyanine green uptake was analysed by treating living cells with 100 µg/ml Cardiogreen (Sigma) for 30 minutes at 37 °C. Bright-field images were acquired with a Leica DM IL LED microscope.

### Antibodies and reagents

All antibodies used in this study were obtained commercially: ApoB (ab31992, Abcam), ApoE (ab52607, Abcam), α-Tubulin (T6074, Sigma), HNF4α (sc-6556, Santa Cruz), GATA-4 (sc-1237, Santa Cruz), Oct3/4 (sc-9081, Santa Cruz), SR-BI (NB400–104, Novus Biologicals), AFP (A8453, Sigma), claudin-1 (ABN-H00009076-M01–100, Biozol), CD81 (clone M38^63^), albumin (A6684, Sigma), occludin (33–1500, Life Technologies), nuclear RED (DRAQ5) (65–0880–92, eBioscience), HRP-conjugated secondary antibodies (Jackson ImmunoResearch), and Alexa 488- and Alexa 594-secondary antibodies (donkey, IgG (H + L), Life Technologies). 60% iodixanol was obtained from Progen (OptiPrep, Axis Shield). If not stated otherwise, cell culture media and supplements were purchased from Gibco/Life Technologies or Sigma and fine chemicals from AppliChem or Sigma.

### HCV RNA *in vitro* transcription and production of viral stocks

For the production of viral stocks, HCV RNA was generated by *in vitro* transcription as described before^58,61^. Briefly, plasmids were linearised, treated with Mung Bean nuclease, and *in vitro* transcribed using the MegaScript T7 kit (Ambion). For transfection, 4 × 10^6^ Huh7.5 cells suspended in 400 µl cytomix buffer (120 mM KCl, 5 mM MgCl_2_, 0.15 mM CaCl_2_, 2 mM EGTA, 1.9 mM ATP, 4.7 mM GSH, 25 mM HEPES, 10 mM potassium phosphate buffer, pH 7.6) were mixed with 10 µg HCV RNA and pulsed at 260 V/950 µF with the Gene Pulser II (Biorad). Culture supernatant of electroporated cells was used to infect naïve Huh7.5 cells for production of viral stocks. Supernatant was harvested, filtered, and either directly aliquoted or concentrated using polyethylene glycol (PEG-8000). Titration of viral stocks was assessed by determination of the 50% tissue culture infectious dose (TCID_50_) by serial limiting dilution on Huh7.5 cells stably expressing the HCV reporter RFP-NLS-IPS^61,64^.

### Production of lentiviral particles and transduction

Lentiviral particles were produced using 293 T cells as described before^61^. Cells were transfected with the shRNA-encoding pSicoR-MS1-Puro vector, a packaging vector (pCMVΔR8.91) and an envelope vector expressing the vesicular stomatitis virus (VSV-G)-glycoprotein (pMD.G). Lentiviral particles were concentrated by ultracentrifugation. Infectious titers were determined by transduction of Huh7 or Huh7.5 cells with serial dilutions of viral stocks, following selection with puromycine 3 days post transduction.

### Gaussia luciferase assays

For analysis of HCV infection, iHLCs and Huh7 cells were incubated with viral stocks of the Jc1^p7-GLuc-2A-NS2^ reporter strain. iHLCs were infected by spin inoculation. 1 day post infection (dpi), cells were washed twice with PBS and once with medium. The media wash was kept as T = 0 and the cell supernatant was harvested every day. For knockdown experiments, iPSCs were differentiated in 12-well plates and transduced with lentiviral stocks on day 12 and day 13 of differentiation in presence of 4 µg/ml polybrene (Sigma). For JAKi-experiments, iPSCs were differentiated in 12-well plates and Huh7 cells were seeded to 96 well plates. 1 h prior to infection, cells were pre-treated with 10 µM JAK-inhibitor (InSolution JAKI Inhibitor, Merck). Media and treatment was renewed daily. Culture supernatant was mixed 1:1 with 2 × Renilla Luciferase Lysis Buffer, inactivated for 1 h at room temperature, and luciferase activity was determined with the Renilla Luciferase Assay System (Promega) on a plate reader (Infinite M200, Tecan).

### Data analysis

For statistical analysis, we used R^65^, RStudio^66^, and GraphPadPrism (GraphPad Software, Inc). Statistical analysis was performed using unpaired two-tailed Welch’s t-test, and in case of normalised data one sample t-test.

### Data availability

The datasets generated during this study are available from the corresponding author on reasonable request.

## Electronic supplementary material

Full-length western blots
