# Supplementary material for: Functional innate immunity restricts Hepatitis C Virus infection in induced pluripotent stem cell–derived hepatocytes
Source: Sci Rep. 2018 Mar 1;8:3893. doi: 10.1038/s41598-018-22243-7 (PMC5832748; doi:10.1038/s41598-018-22243-7)
Supplement: Supplementary file 1 — Full-length western blots [file 41598_2018_22243_MOESM1_ESM.pdf]

Functional innate immunity restricts Hepatitis C Virus infection in induced pluripotent stem cell–derived hepatocytes

Anja Schöbel[1], Kathrin Rösch[1], and Eva Herker[1,*]

[1] Heinrich Pette Institute, Leibniz Institute for Experimental Virology, Hamburg, Germany

*Address correspondence to: Eva Herker, Heinrich Pette Institute, Leibniz Institute for Experimental Virology, Martinistrasse 52, 20251 Hamburg, Germany, E-mail: eva.herker@leibniz-hpi.de

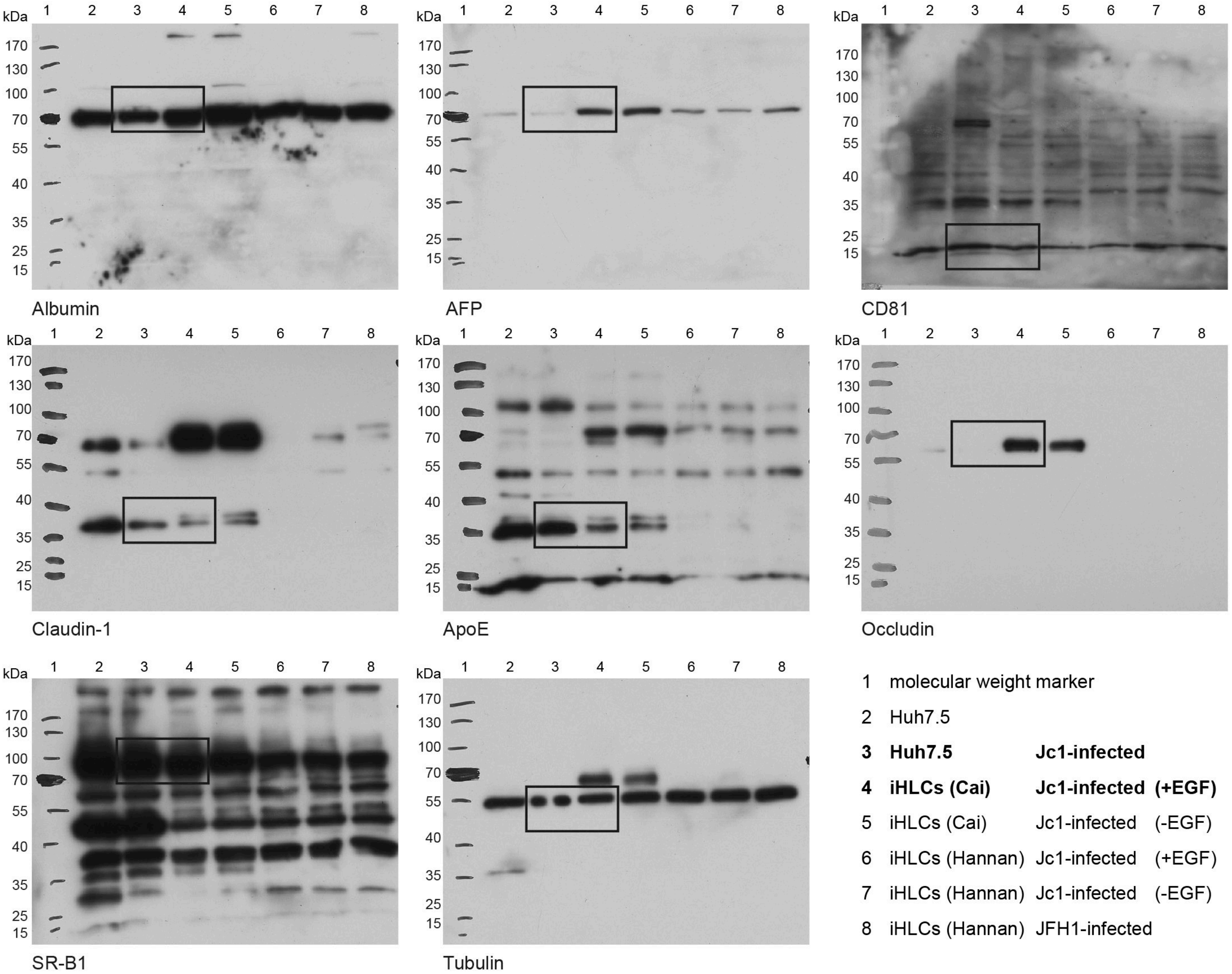

Supplementary Figure 1: Full-length western blots of HCV host factors, related to Figure 1

Western blot analysis of HCV host factor expression in iHLCs and Huh7.5 cells. Equal amounts of protein were subjected to SDS-PAGE followed by western blotting and incubation with the indicated antibodies. Samples were run in duplicate on two SDS gels with the same loading scheme (lanes 1–8). 1: molecular weight marker; 2: Huh7.5; 3: Jc1-infected Huh7.5; 4: Jc1-infected iHLCs (+EGF); 5: Jc1-infected iHLCs (-EGF); 6: Jc1-infected iHLCs (+EGF); 7: Jc1-infected iHLCs (-EGF); 8: JFH1-infected iHLCs. iHLCs in lane 4 and 5 were differentiated following the protocol we used in this publication [1], iHLCs in lane 6–8 were differentiated using a different protocol [2]. Bands shown in Figure 1d are marked with a blacked box. We used the following commercial antibodies: occludin [3] (clone OC-3F10, # 33-1500, Life Technologies), claudin-1 [4] (clone 1C5-D9, # H00009076-M01, Abnova/Biozol), SR-BI [5] (# NB400-104, Novus Biologicals), ApoE [6] (clone EP1374Y, # ab52607, Abcam), AFP [7] (A8453, Sigma), α-Tubulin [8] (clone B-5-1-2, # T6074, Sigma), and HRP-conjugated secondary antibodies (Jackson ImmunoResearch), as well as CD81 (clone M38) [9]. Full-length blots are related to Figure 1D.

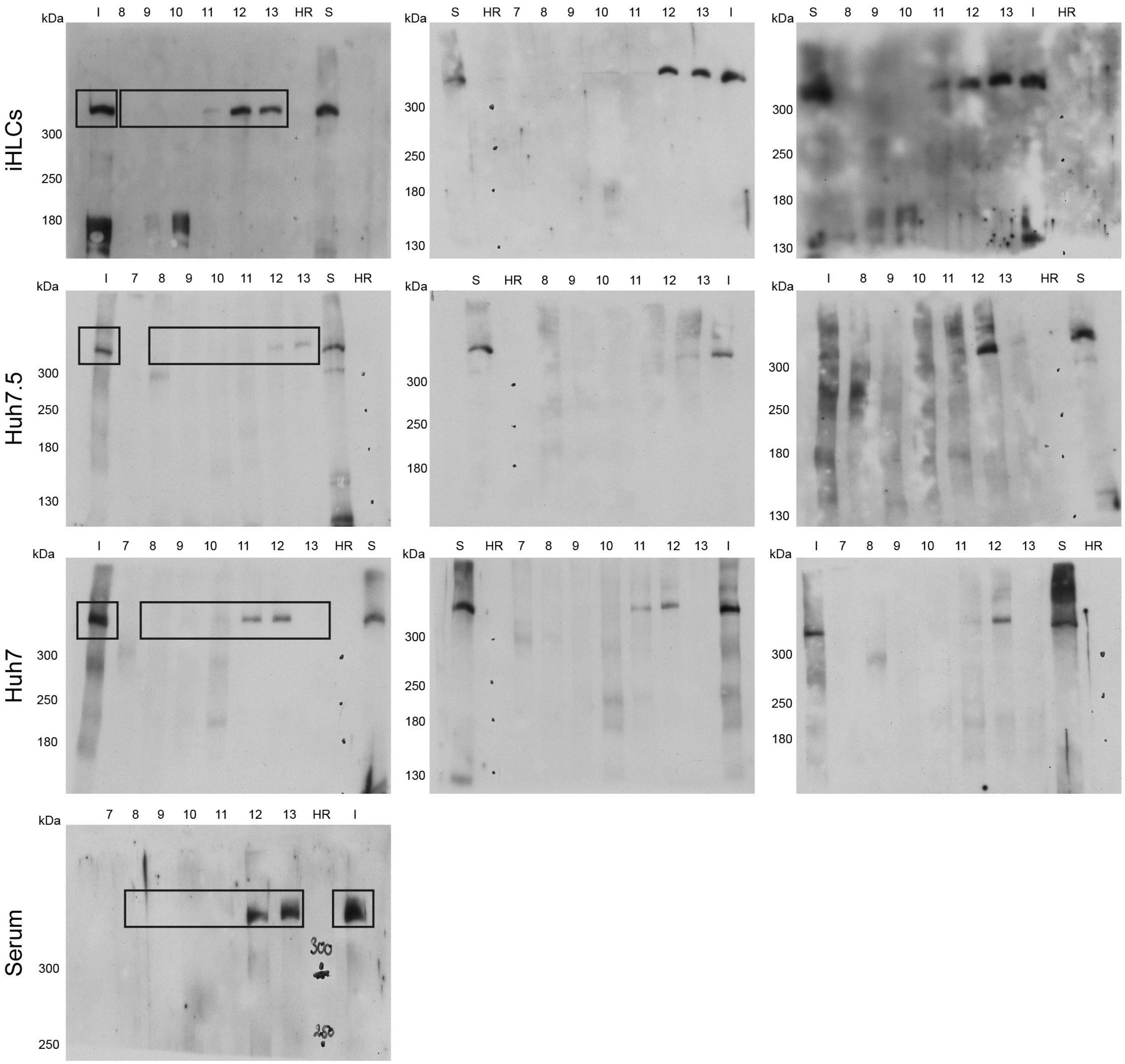

Supplementary Figure 2: Full-length western blots of ApoB secretion, related to Figure 2

For characterization of lipoproteins secreted by iHLCs, Huh7, and Huh7.5 cells, cell culture supernatants and human serum were subjected to linear density gradients. Western blot analysis of density gradient fractions using a commercial antibody directed against ApoE [6] (clone EP1374Y, # ab52607, Abcam). Shown are all full-length blots displayed in Figure 2a (marked with a black box) and used for the quantification in Figure 2b. Lanes are marked with I: input; 7–13: gradient fractions from bottom to top; HR: high range molecular weight marker, S: serum.

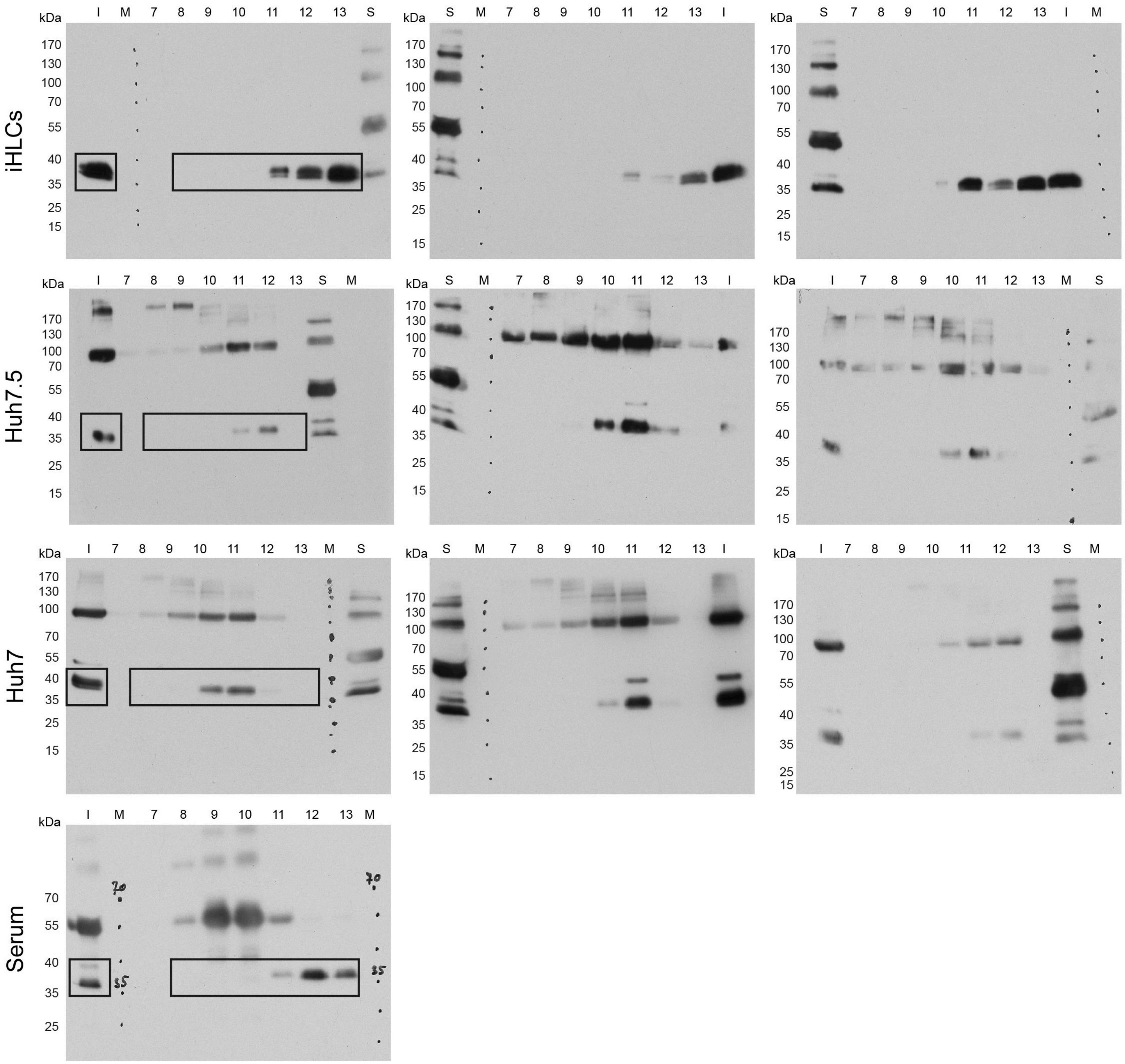

Supplementary Figure 3: Full-length western blots of ApoE secretion, related to Figure 2

For characterization of lipoproteins secreted by iHLCs, Huh7, and Huh7.5 cells, cell culture supernatants and human serum were subjected to linear density gradients. Western blot analysis of density gradient fractions using a commercial antibody directed against ApoB [8] (# ab31992, Abcam). Shown are all full-length blots displayed in Figure 2a (marked with a black box) and used for the quantification in Figure 2b. Lanes are marked with I: input; 7–13: gradient fractions from bottom to top; M: molecular weight marker, S: serum.

Supplementary References

1 Cai, J. *et al.* Protocol for directed differentiation of human pluripotent stem cells toward a hepatocyte fate. *StemBook*, doi:doi/10.3824/stembook.1.52.1 (2012).

2 Hannan, N. R., Segeritz, C. P., Touboul, T. & Vallier, L. Production of hepatocyte-like cells from human pluripotent stem cells. *Nat. Protoc.* **8**, 430-437 (2013).

3 Sourisseau, M. *et al.* Temporal analysis of hepatitis C virus cell entry with occludin directed blocking antibodies. *PLoS Pathog.* **9**, e1003244, doi:10.1371/journal.ppat.1003244 (2013).

4 Harris, H. J. *et al.* CD81 and claudin 1 coreceptor association: role in hepatitis C virus entry. *J. Virol.* **82**, 5007-5020, doi:10.1128/JVI.02286-07 (2008).

5 Scarselli, E. *et al.* The human scavenger receptor class B type I is a novel candidate receptor for the hepatitis C virus. *EMBO J.* **21**, 5017-5025 (2002).

6 Liefhebber, J. M., Hague, C. V., Zhang, Q., Wakelam, M. J. & McLauchlan, J. Modulation of triglyceride and cholesterol ester synthesis impairs assembly of infectious hepatitis C virus. *J. Biol. Chem.* **289**, 21276-21288, doi:10.1074/jbc.M114.582999 (2014).

7 Si-Tayeb, K. *et al.* Highly efficient generation of human hepatocyte-like cells from induced pluripotent stem cells. *Hepatology* **51**, 297-305, doi:10.1002/hep.23354 (2010).

8 Herker, E. *et al.* Efficient hepatitis C virus particle formation requires diacylglycerol acyltransferase-1. *Nat. Med.* **16**, 1295-1298, doi:10.1038/nm.2238 (2010).

9 Fritzsching, B. *et al.* Release and intercellular transfer of cell surface CD81 via microparticles. *J. Immunol.* **169**, 5531-5537 (2002).
